# I think, therefore I hit: EEG parameters predict performance in complex cognitive tasks

**DOI:** 10.3389/fnrgo.2026.1894934

**Published:** 2026-09-23

**Authors:** Anneke Hamann, Julia Schön, Nils Carstengerdes

**Affiliations:** Institut für Flugführung, Deutsches Zentrum für Luft- und Raumfahrt e.V. (DLR), Braunschweig, Germany

**Keywords:** aviation, cross-task performance prediction, EEG, mental workload, n-back, operator state, Space Fortress Game, training

## Abstract

In recent years, operator state assessment has gained much attention in the aviation domain. The ability to assess or even predict performance from behavioral or physiological data offers the possibility to tailor training schedules and assistance systems to the human operators' needs. Yet, to date, there is still no valid or reliable method for such an assessment. In the present study, we present evidence that brain activity recorded via electroencephalography (EEG) can be used to predict complex cognitive task performance. 26 participants performed multiple rounds of a dual n-back task and the complex Space Fortress Game while their brain activity was recorded via EEG. Results from multiple linear regression analyses indicate that Space Fortress Game performance could be predicted reasonably well from EEG Power Spectral Density parameters from the same game, a previous game and from the dual n-back task. Our findings show that it is possible to use data from task training and even from dissimilar, less complex tasks to predict complex task performance (in offline analyses after data collection). They also underline the utility of using EEG measurements for operator state monitoring.

## 1 Introduction

The aviation domain is characterized by complex, cognitively demanding tasks for human operators as well as a constant trend of increasing automation. Modern aircraft are a good example for this, transitioning from first rudimentary aircraft with fully manual flight control to almost fully automated navigation and landing in less than 70 years (Billings, 1991). In recent years, a new group of operators has entered the aviation domain. With the emergence of unmanned aerial vehicles (UAVs), humans are tasked with controlling one to several to whole swarms of semi-autonomous UAVs. These UAV operators have similar navigation, planning and decision-making tasks as aircraft pilots, but with a different level of complexity and different information needs (Hocraffer and Nam, 2017; Huttner and Friedrich, 2023). Because motor tasks that require physical strain are often the easiest to automate, pilots and UAV operators increasingly assume the role of planners, decision-makers and observers who monitor automation that carries out tasks (Mosier et al., 2013; Hocraffer and Nam, 2017). This also means that the humans retain the majority of complex cognitive aviation tasks as well as the new task of understanding, monitoring and correcting the automation (Bainbridge, 1983; Sheridan, 1999, 2012). Moreover, while acquired motor skills are usually retained for years, cognitive skills degrade much more quickly, needing constant training and refreshing (Patrick, 1992).

Pilot training is highly regulated and consists of a mixture of initial, transition and recurrent trainings of both knowledge and practical skills (Feary, 2018). Competency-based training, i.e., training schedules and needs based on demonstrated skills, allows some flexibility and can make trainings more efficient (Kearns et al., 2016). Still, these trainings are time- and resource-consuming (Zarlengo, 2025). For UAV operators, such regulations are not yet in place. Moreover, even competency-based training is mainly anchored on observable behavior. But many steps leading to observable performance are purely cognitive and cannot be observed, such as attention direction, information gathering, interpretation or the application of prior knowledge to current problems (Pritchett and Hansman, 2000). Bad performance can be based on excellent information processing and decision-making, and good performance can occur despite problems in previous cognitive steps (Pritchett and Hansman, 2000). Thus, performance as an outcome may not be sufficient to explain gaps in the operators' knowledge or skills. In addition, the unobservable parts of a task gain importance in highly automated tasks where pilots or UAV operators mainly monitor and make decisions based on the information gathered from the automation (Mosier et al., 2013; Hocraffer and Nam, 2017). Consequently, trainings could be improved by information about the cognitive components of a task and the operators' cognitive state while training, such as their level of cognitive demand or attention distribution.

In recent years, operator state assessment has gained much attention in aviation research (Borghini et al., 2014; van Weelden et al., 2022; Boumann et al., 2023; Hamann et al., 2026). The aim of such monitoring is to continuously assess the operators' cognitive state and evaluate their ability to fulfill their tasks by means of measuring various behavioral and physiological metrics in real time. Research has shown that it is possible to use brain activity to detect operators' drowsiness (Hussein et al., 2023), mental workload on multiple levels (Hamann and Carstengerdes, 2022), and even their spatial attention to visual information (Ladouce and Dehais, 2024) via portable neurophysiological measurements like electroencephalography (EEG) or functional near-infrared spectroscopy (fNIRS). The next logical step is to use this information to predict performance and reveal early signs of inattention, sleepiness or excessive cognitive demand before the operators' performance declines.

Over the years, there have been countless attempts estimating or predicting changes in cognitive states such as mental workload, drowsiness or engagement within the same task (Ur Rahman et al., 2025; Verkennis et al., 2025; Ji et al., 2026) and across different tasks (Ke et al., 2015; Walter, 2015; Natalizio et al., 2024). Interestingly, less attention has been paid to the prediction of task performance from brain activity. The paradigms usually involve performance prediction based on concurrent or past brain activity in the same task (Lin et al., 2005; Ferrez and Del R Millan, 2008; Stikic et al., 2011; Apker et al., 2013; Walter et al., 2014; Ayaz et al., 2019; Pušica et al., 2024; Castro et al., 2026). To our knowledge, however, the literature on cross-task performance prediction is still surprisingly sparse (Stikic et al., 2014; Fernandez-Vargas et al., 2021).

There is evidence that performance in complex cognitive and psychomotor tasks can be predicted by brain activity during the early stages of training (via functional magnetic resonance imagery [fMRI]; Vo et al., 2011; Mathewson et al., 2012), or by corresponding brain activity during task completion, as well as by brain activity during rest (via fNIRS; Chenot et al., 2021). The authors of these studies used a paradigm called the Space Fortress Game (SFG), a single-player computer game combining psychomotor with working memory and action inhibition skills that was specifically developed to study complex skill acquisition (Logie et al., 1989; Mané and Donchin, 1989; Donchin, 1995). This task has been shown to correlate with intelligence tests (Rabbitt et al., 1989; Lee et al., 2015), and to be of interest in assessing individual skill acquisition (Towne et al., 2016) and workload-dependent strategy change (Moon et al., 2013). Because of its need for good psychomotor skills, working memory capacity, visual demands, situation assessment and strategy adaption, the SFG transfers well to actual flying skills (Hart and Battiste, 1992; Gopher et al., 1994). This makes the SFG an excellent task both for studying the effects of task practice on brain activity, and as an approximation for complex cognitive tasks like pilots' or UAV operators' tasks.

Thus, in the current study, we wanted to expand the current state of research by analyzing which EEG parameters (specifically, frequency domain Power Spectral Density PSD) could predict complex multitasking performance. Specifically, we wanted to see if task performance could be predicted by EEG activity from previous runs of the same task and across tasks, i.e., from a different, less complex working memory task (predictive value of training), and from concurrent brain activity during task completion (concurrent operator state assessment). We used the SFG task as the complex task due to its relatedness to pilots' tasks (Hart and Battiste, 1992; Gopher et al., 1994) and a dual n-back task as the dissimilar second task because of a strong association between working memory and SFG performance (Logie et al., 1989). We first analyzed how different EEG parameters were influenced by practice and task difficulty in order to check the success of our manipulation and to down-select runs for further analyses. We then computed stepwise both-directions regressions to see which EEG parameters would predict SFG performance best.

## 2 Material and methods

### 2.1 Sample

27 volunteers were recruited for this study (limited by facilities availability). Due to a recording issue, the data of one participant had to be excluded, resulting in a sample of 26 participants: 18 male and eight female, aged 20 to 54 (*M* = 28.4, *SD* = 8.1). Inclusion criteria were: age 18 to 59 years; native German language skills; higher education qualification (A-levels or equivalent, or higher education degree); right-handedness; normal hearing, normal or corrected-to-normal vision and normal color perception; no diagnosed neurological conditions. The participants received information about the study, and provided written informed consent. The study was approved by the DLR ethics commission “Geschäftsstelle Forschungsethik” (Approval No. 21/24) and conducted in accordance with the Declaration of Helsinki.

### 2.2 Tasks and material

#### 2.2.1 Dual n-back task

The n-back task is a well-known and widely used working memory task originally developed by Kirchner (1958). In the original n-back paradigm, participants are presented with a sequence of visual or auditory stimuli, such as letters, numbers or spatial positions, and need to indicate if the present stimulus is the same as the one *n* prior (Kirchner, 1958). In this way, working memory load (and thus task difficulty) can be increased stepwise by increasing *n* (Braver et al., 1997), resulting in a monotonous decrease in performance as well (Bopp and Verhaeghen, 2020). Over the years, a variety of different n-back tasks has been developed (Owen et al., 2005). One example is the dual n-back task that presents two types of stimuli in parallel and requires participants to memorize and respond to two stimuli per trial. In the present study, we used a dual n-back task that combines auditory and spatial memory. We used the “Dual n-Back: Brain Training” iPad app (Lindware OÜ, Tallin, Estonia) and presented the game on an Apple iPad 9th generation (10.2”). In this n-back version, participants initially see a black screen with a gray fixation cross which the participants were instructed to fixate on. In each trial, participants hear a single English letter pronounced by a female computer voice (auditory stimulus) and simultaneously see one tile within a 3 x 3 grid flashing yellow (spatial stimulus). Upon the first required reaction, two buttons appear on the lower part of the screen which the participant needs to press to indicate a matching sound (left button) or position (right button). Upon starting a run, a countdown from three is presented before the first trial. Each run ends automatically after the last trial and the participant receives immediate feedback on their performance (% correct). Each trial lasts approx. 3 s. Each run comprises *n* trials to be memorized in the beginning plus 20 trials requiring a reaction. This means, a 1-back condition comprises 21 trials (approx. 63 s), a 2-back 22 trials (approx. 66 s) and a 3-back 23 trials (approx. 69 s) etc. In each run and across conditions, approx. 30% of all auditory and 30% of all visual stimuli are targets. The targets are randomly distributed across the run, resulting in a random combination of auditory-only, visual-only or audiovisual target trials. For this experiment, we used three difficulty levels, 1-, 2-, and 3-back. Because of the short duration of one run, two runs of the same difficulty were presented consecutively and combined into one block. After completing a block, the difficulty level was changed. Each difficulty level was presented twice in a pseudo-randomized order, i.e., never the same level in direct succession, resulting in six task blocks (i.e., 3 difficulty levels x 2 blocks) of approx. 2 min length each.

#### 2.2.2 Space Fortress Game

The SFG is a game designed to study complex skill acquisition (Mané and Donchin, 1989; Donchin, 1995). In the game, the participant steers a spaceship and tries to destroy an enemy ship, the “fortress” while simultaneously identifying and destroying mines, avoiding being hit and capturing bonuses. Details on the basic game principle can be found in Boot et al. (2010) and Chenot et al. (2021). In its original version, SFG comprises vector graphics images. For the present study, we used the same version of the game as Chenot et al. (2021), a version with more modern graphic elements implemented in Python 2.7 (https://github.com/CogWorks/SpaceFortress), see Figure 1. The game was played on a computer with a screen (2560 x 1440 pixels, 27 inch) and keyboard in front of the participant. The relevant keys for moving the space ship, for shooting, and for collecting bonuses were marked with colored sticky dots. In deviation from the game parameters used by Chenot et al. (2021), we set the duration of one game to 2 min in order to match the duration of one n-back block. The payout for the bonuses was corrected accordingly to 25 shots. Additionally, the maximum number of shots a player could have at any given time was capped at 50. All audio except bonus feedback was removed from the game. In total, six games were played. In the beginning of each run, the participant had 50 shots. The participant received immediate feedback about their performance at the end of each run (number of player deaths, destroyed mines, destroyed fortresses, and total points awarded).

**Figure 1:**
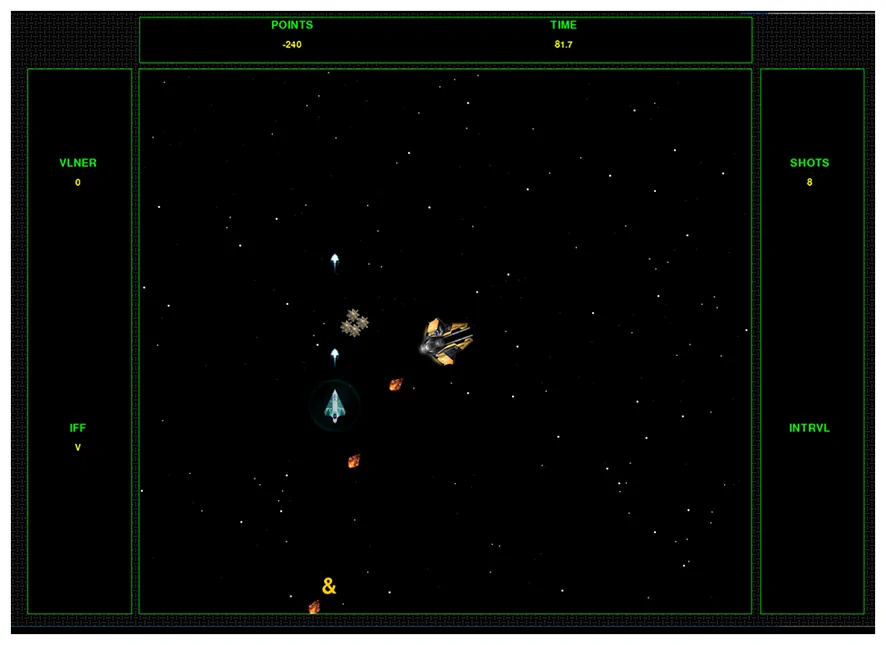
The Space Fortress Game. The participant's ship (blue, center-left) is in the process of firing on a mine (gray) and avoiding hits from the fortress (yellow-black, center). The margins of the screen show game information like available resources, time left, and current score.

#### 2.2.3 Subjective mental workload

We collected subjective MWL ratings with the NASA-TLX including the original weighting procedure (Hart and Staveland, 1988). The NASA-TLX is a 6-dimension MWL rating instrument with a range from 0 to 100. Higher numbers indicate higher demand. In order to administrate it, we used the NASA-TLX iPad app version 1.0 (NASA Ames, Moffett Field, USA). The NASA-TLX was administered after each n-back block (i.e., after two runs of the same difficulty) and after each SF game. The weighting procedure was done once for the n-back task and a second time for the SF game, each after the first block/game. The NASA-TLX was presented on the same iPad as the n-back task.

#### 2.2.4 EEG data recording

EEG data were recorded with a LiveAmp-32 device and in BrainVision Recorder 1.26 (Brain Products GmbH, Gilching, Germany). All impedances were kept at ≤ 10 kΩ. The initial sampling rate was 500 Hz. 26 Ag/AgCl active EEG electrodes were positioned according to the 10–20 system with online reference at FCz. Six electrodes (FT9, FT10, T7, T8, TP9, TP10) were left out so participants could wear glasses during data acquisition.

### 2.3 Procedure

The experiment lasted around 2 to 2.5 h. Upon entering the laboratory, the participants received information about the experiment, gave written informed consent, and answered a demographic questionnaire. They were then equipped with the EEG system, followed by the two experimental tasks (dual n-back, SFG) including instructions and practice sessions. The order of the experimental tasks was balanced between participants. For the n-back task, the participants were asked to practice each n-back level twice (1-, 2-, 3-back in ascending difficulty), but if they had an accuracy of 80 % or better on the first try, they were allowed to move to the next difficulty level immediately. Following the practice session, the participants performed the n-back task and rated their subjective MWL after each block. For the SFG, the participants underwent stepwise training aimed at building competences for the different aspects of the game, in a simplified and shortened version of the part-task training implemented by Lee et al. (2012). In total, each participant played four practice games with focus on (1) ship navigation; (2) hitting the fortress and destroying mines; (3) collecting bonuses; (4) all tasks combined. They then performed six SFG rounds, each followed by a NASA-TLX rating. Once both experimental tasks were finished, the participants were relieved of the EEG equipment, given the possibility to wash their hair, thanked and seen off.

### 2.4 Data processing and analysis

#### 2.4.1 Subjective mental workload and performance

Weighted NASA-TLX scores for the n-back and SFG were computed by multiplying the ratings on each of the six dimensions with their assigned weights, then dividing by 15 and averaging (Hart, 1986).

n-back performance was calculated as % correct reactions per block by the app. A correct reaction is defined as a correct button press in case of a stimulus match and a correct inaction in case of no match. In case both sound and position matched, the trial is only counted as correct if both buttons are pressed.

For the SFG, five performance metrics were used, the overall high score computed by the game and four metrics were adapted from previous research (Chenot et al., 2021): collisions per min (CPM), fortress hits per min (FPM), mines destroyed (MD) and bonuses captured (BC), see Table 1. While CPM and FPM mostly depend on player behavior, MD and BC also depend on the number of spawned mines and available bonuses per game, as these are generated randomly by the software. Thus, we chose to qualify the latter not on game time but on available chances in the game.

**Table 1:** Four performance metrics calculated for the Space Fortress Game and their connection to the game tasks and rules.

Metric	Sub-task	Game rule	Score calculation
collisions per min (CPM)	controlling the ship	•avoid being hit by fortress •avoid being hit by mine •avoid colliding with fortress •avoid flying outside of game borders	sum of events, divided by game min
fortress hits per min (FPM)	destroying the fortress	•hit and destroy the fortress	sum of successful fortress hits, divided by game min
mines destroyed (MD)	destroying the mines	•identify and destroy mines	number of successfully destroyed mines, divided by all spawned mines
bonuses captured (BC)	capturing bonuses	•capture bonuses	number of successfully captured bonuses, divided by all possible bonuses

#### 2.4.2 EEG data

EEG data were pre-processed using the BrainVision Analyzer 2.2 (Brain Products GmbH, Gilching, Germany). The data were down-sampled from 500 to 256 Hz to avoid zero-padding in the following Fast Fourier Transformations. No excessively noisy channels were identified and all channels were re-referenced to average and a 4th order IIR bandpass filter between 0.5–40 Hz was applied. An additional notch filter at 50 Hz was added to remove remaining line noise from the recording environment. The following pre-processing steps were undertaken separately for the n-back and SFG tasks to account for different eye and movement artifacts due to the task characteristics. Ocular artifacts were corrected using the Ocular Correction Independent Component Analysis (ICA) implemented in the software: a Restricted Infomax ICA was applied on the whole data, with electrode Fp1 for identifying vertical and horizontal eye activity (in two instances, Fp2 was used instead due to occasional high noise in Fp1). The pre-trained Slope Algorithm implemented in the software was used for blink detection as well as squared correlations with the respective channel, set to 30 % deletion. The algorithm was set to semi-automatic inspection, i.e., components were recommended for removal by the software based on the computed energy values, and additional visual inspection was performed to select or de-select components. In most datasets and task, two components were removed (one for horizontal, one for vertical activity) with the exception of four instances of the SFG and one instance of the n-back task in which three ICA components were removed. Further movement artifacts were removed by semi-automatic inspection based on threshold values of gradient (20 μV), difference (200 μV), amplitude (−200 to 200 μV) and low activity (0.5 μV) per electrode. The thresholds were adapted as necessary if the initial criteria resulted in too much or too little removal (five times for the n-back task, two times for the SFG task). In addition, a visual inspection was performed to identify artifacts missed by the algorithm. On average, in the n-back task, 0.11 % of data were removed per dataset; and 0.09 % in the SFG. The n-back task data were segmented first into each run, based on start and end markers, and then further segmented into epochs of 2 s with 0.5 s overlap. For the following steps, each two consecutive runs of the same difficulty were combined into one block. The SFG data were segmented into games, based on start and end markers, then further segmented into epochs of 2 s with 0.5 s overlap. For both tasks, the data were then transformed into the frequency domain using a Fast Fourier Transformation with a Hanning window with 10 % overlap, and averaged for each block or game, respectively (this approach approximates Welch's procedure in the Analyzer 2 software).

The data were exported as Power Spectral Density (PSD) raw sum (μV^2^/Hz). The following EEG parameters were exported: for analysis of single bands, theta band (4–8 Hz) for the frontal electrodes Fz, F3, F4, as well as alpha band (8–13 Hz) and beta (13–30 Hz) band for the parietal electrodes Pz, P3, P4. In addition, we included two well-known EEG indices, the workload-related Task Load Index TLI (Smith et al., 2001) and the attention-associated Engagement Index EI (Pope et al., 1995). The TLI is defined as the ratio of theta power (6–7 Hz) at Fz and alpha power (8–12 Hz) at Pz. The EI is defined as beta 13–22 Hz / (alpha 8–13 Hz + theta 4–8 Hz), each averaged across the electrodes Cz, Pz, P3 and P4.

All parameters were checked for normal distribution using the Shapiro-Wilk test. Since significant deviations were found, the data were ln-transformed per band and electrode to account for skewness. TLI and EI were computed on the ln-transformed data.

#### 2.4.3 Analysis strategy

All analyses were conducted in RStudio 2025.05.1 (R 4.5.1). In a first step, we analyzed how the task characteristics (task difficulty, repetition) influenced EEG parameters, task performance and subjective MWL. This served as a manipulation check and quantification of possible learning effects, and informed our decision on which data to include in the regression analyses described below. For this first analysis, we computed mixed-effects models using the *lmer* function of the *lmerTest* package, followed by analyses of variance (ANOVAs) separately for the n-back and SFG task. Dependent variables (DV) were: frontal theta at Fz, F3, F4, parietal alpha at Pz, P3, P4, parietal beta at Pz, P3, P4, TLI and EI; NASA-TLX; % correct (n-back); or score, CPM, FPM, MD, BC (SFG). For the n-back task, the model included the factors n-back level (1-, 2-, 3-back) and run (first, second), their interaction and a random intercept for the participant: “DV ~ level ^*^ run + (1|participant)”. For the SFG task, the model included only the factor game number and a random intercept for the participant: “DV ~ game + (1|participant)”. Significant effects were followed by *post-hoc* pairwise comparisons using the *emmeans* function including *p*-value adjustment for multiple comparisons via the Tukey method.

For the main analyses, the prediction of complex task performance via EEG parameters, we first wanted to select the most appropriate of the several performance metrics of the SFG. To further analyze the relationship between the performance metrics, we calculated repeated-measures correlations between the overall score and sub-scores using the *rmcorr* function (Bakdash and Marusich, 2017, 2019). Based on these analyses and previous literature (Chenot et al., 2021), we decided to use the overall performance score as DV instead of the sub-scores. We then conducted both-directions regression analyses with the SFG scores as DV and the eleven extracted EEG parameters from either the n-back tasks, from previous SFG runs (predictive value of training) or from the same SFG run (concurrent operator state assessment) as predictors. We used the *step* function of the *stats* package, which selects the most informative, yet most parsimonious model based on the Akaike information criterion (AIC) (Burnham and Anderson, 2004; Murtaugh, 2009).

## 3 Results

### 3.1 Influence of task characteristics on subjective mental workload, performance and EEG parameters

In the n-back task, frontal theta power and the TLI increased with increasing task difficulty. Parietal alpha and beta power and the EI decreased with increasing task difficulty but increased between runs. The effects on the EEG parameters were most prominent for 1-back vs. the two more difficult levels. Performance decreased with increasing task difficulty while NASA-TLX ratings increased. In sum, this indicates increasing mental workload with increasing task difficulty. See Table 2 for details and descriptives, and S1 for *post-hoc* tests.

**Table 2:** Descriptive values and results of the mixed-effects ANOVAs for the n-back task.

Parameter	Descriptive values (*M, SD*) per condition (n-back level, run)						ANOVA results		
	1, 1	2, 1	3, 1	1, 2	2, 2	3, 2	Level, *F(2, 125), p-value*	Run, *F(1, 125), p-value*	Level ^*^Run, *F(2, 125), p-value*
Theta Fz	2.36 (0.43)	2.39 (0.44)	2.38 (0.44)	2.36 (0.45)	2.43 (0.44)	2.39 (0.47)	6.01 (0.003^*^)	2.18 (0.142)	0.78 (0.459)
Theta F3	2.05 (0.59)	2.05 (0.51)	2.04 (0.61)	2.06 (0.59)	2.09 (0.59)	2.05 (0.61)	1.17 (0.314)	1.50 (0.223)	0.87 (0.420)
Theta F4	1.98 (0.40)	1.96 (0.38)	1.94 (0.39)	1.94 (0.42)	1.98 (0.41)	1.93 (0.40)	3.78 (0.025^*^)	1.02 (0.314)	1.95 (0.147)
Alpha Pz	1.84 (0.75)	1.80 (0.68)	1.79 (0.73)	1.93 (0.82)	1.87 (0.77)	1.85 (0.74)	4.37 (0.015^*^)	14.52 (<0.001^*^)	0.28 (0.754)
Alpha P3	1.92 (0.74)	1.86 (0.69)	1.89 (0.70)	2.00 (0.79)	1.92 (0.76)	1.92 (0.73)	5.28 (0.006^*^)	10.33 (0.002^*^)	0.69 (0.501)
Alpha P4	2.00 (0.79)	1.92 (0.72)	1.93 (0.77)	2.04 (0.84)	2.00 (0.81)	1.97 (0.78)	5.92 (0.003^*^)	8.80 (0.004^*^)	0.73 (0.484)
Beta Pz	1.81 (0.50)	1.76 (0.47)	1.75 (0.51)	1.86 (0.53)	1.78 (0.50)	1.78 (0.49)	20.78 (<0.001^*^)	11.31 (0.001^*^)	0.89 (0.413)
Beta P3	2.22 (0.47)	2.18 (0.43)	2.21 (0.47)	2.27 (0.48)	2.20 (0.48)	2.18 (0.46)	4.40 (0.014^*^)	1.26 (.265)	2.34 (0.101)
Beta P4	2.22 (0.46)	2.22 (0.41)	2.22 (0.46)	2.29 (0.45)	2.23 (0.47)	2.23 (0.48)	1.73 (.181)	4.33 (0.039^*^)	1.94 (0.148)
TLI	0.61 (0.24)	0.66 (0.28)	0.65 (0.30)	0.62 (0.28)	0.66 (0.25)	0.64 (0.28)	3.56 (0.031^*^)	0.02 (0.879)	0.29 (0.750)
EI	0.45 (0.08)	0.44 (0.07)	0.45 (0.08)	0.46 (0.08)	0.44 (0.08)	0.45 (0.08)	6.52 (0.002^*^)	0.39 (0.535)	1.83 (0.165)
NASA-TLX	37 (15)	61 (10)	66 (9)	40 (21)	53 (13)	65 (13)	85.31 (<0.001^*^)	1.08 (0.300)	3.38 (0.037^*^)
% correct	98 (4)	88 (6)	79 (6)	98 (2)	91 (5)	81 (6)	207.12 (<0.001^*^)	5.13 (0.025^*^)	1.16 (0.316)

Significance threshold α = 0.05.

Significant results marked^*^.

In the SFG task, the number of games played only influenced two parietal alpha parameters and the TLI as well as some of the performance metrics. Alpha power at Pz and P3 increased slightly with the number of games played. The TLI decreased instead. The overall score, FPM, MD and BC increased slightly with the number of games played. *Post-hoc* tests revealed that significant changes were mainly between the first two and the later games, indicative of a learning curve during the first two games. See Table 3 for details and descriptives, and S2 for *post-hoc* tests.

**Table 3:** Descriptive values and results of the mixed-effects ANOVAs for the SFG task.

Parameter	Descriptive values (*M, SD*) per condition (SFG number)						ANOVA results
	1	2	3	4	5	6	Game, *F(5, 125), p-value*
Theta Fz	2.47 (0.47)	2.52 (0.48)	2.48 (0.47)	2.51 (0.49)	2.47 (0.45)	2.50 (0.50)	1.40 (0.230)
Theta F3	2.15 (0.46)	2.17 (0.42)	2.16 (0.42)	2.20 (0.47)	2.15 (0.42)	2.16 (0.45)	1.44 (0.214)
Theta F4	2.16 (0.48)	2.20 (0.47)	2.14 (0.43)	2.17 (0.47)	2.14 (0.44)	2.15 (0.48)	2.08 (0.072)
Alpha Pz	1.76 (0.55)	1.82 (0.54)	1.82 (0.53)	1.83 (0.56)	1.81 (0.56)	1.83 (0.55)	3.20 (0.009^*^)
Alpha P3	1.67 (0.57)	1.71 (0.57)	1.74 (0.58)	1.74 (0.60)	1.73 (0.60)	1.72 (0.57)	2.72 (0.023^*^)
Alpha P4	1.74 (0.62)	1.77 (0.63)	1.78 (0.61)	1.79 (0.63)	1.80 (0.63)	1.76 (0.64)	1.77 (0.124)
Beta Pz	1.66 (0.36)	1.69 (0.37)	1.69 (0.36)	1.69 (0.38)	1.69 (0.36)	1.70 (0.37)	1.83 (0.113)
Beta P3	1.89 (0.39)	1.91 (0.37)	1.96 (0.38)	1.91 (0.39)	1.94 (0.37)	1.92 (0.39)	1.00 (0.424)
Beta P4	1.94 (0.38)	1.96 (0.38)	2.01 (0.40)	1.98 (0.42)	1.98 (0.39)	1.98 (0.41)	1.68 (0.145)
TLI	0.69 (0.31)	0.73 (0.32)	0.66 (0.32)	0.71 (0.30)	0.68 (0.37)	0.70 (0.33)	1.68 (0.045^*^)
EI	0.37 (0.06)	0.37 (0.06)	0.38 (0.06)	0.37 (0.06)	0.37 (0.06)	0.37 (0.06)	1.79 (0.120)
NASA-TLX	70 (14)	70 (17)	70 (16)	68 (17)	67 (17)	68 (18)	0.85 (0.514)
Score	−161 (509)	−217 (610)	−115 (572)	−38 (451)	35 (582)	71 (470)	3.63 (0.004^*^)
CPM	4.9 (3.2)	5.3 (4.4)	5.0 (4.0)	4.3 (3.3)	4.2 (3.9)	4.1 (3.2)	−0.81 (0.965)
FPM	3.38 (1.85)	3.92 (2.13)	3.96 (2.09)	4.52 (2.72)	5.08 (2.93)	4.83 (2.61)	4.08 (0.002^*^)
MD	0.47 (0.23)	0.44 (0.23)	0.48 (0.22)	0.52 (0.19)	0.57 (0.21)	0.55 (0.25)	2.55 (0.031^*^)
BC	0.29 (0.21)	0.33 (0.25)	0.33 (0.25)	0.37 (0.26)	0.41 (0.22)	0.44 (0.26)	3.37 (0.007^*^)

Significance threshold α = 0.05.

Significant results marked^*^.

### 3.2 Correlations between SFG performance metrics

The repeated-measures correlations between the overall SFG scores and sub-scores across all six games can be found in Table 4 and Figure 2. There was a strong negative association between the score and flight performance (i.e., fewer collisions equal higher scores), a moderate positive association between the players' score and their performance in destroying mines and capturing bonuses, but no meaningful association between the score and hits on the fortress.

**Table 4:** Results of the repeated-measures correlations between SFG score and sub-scores.

Correlation	*r*	df	*p*	95% CI
score ~ CPM	−0.776	129	<0.001^*^	[−0.836, −0.698]
score ~ FPM	0.127	129	0.119	[−0.035, 0.301]
score ~ MD	0.368	129	<0.001^*^	[0.210, 0.508]
score ~ BC	0.411	129	<0.001^*^	[0.258, 0.544]

Significant values marked^*^.

**Figure 2:**
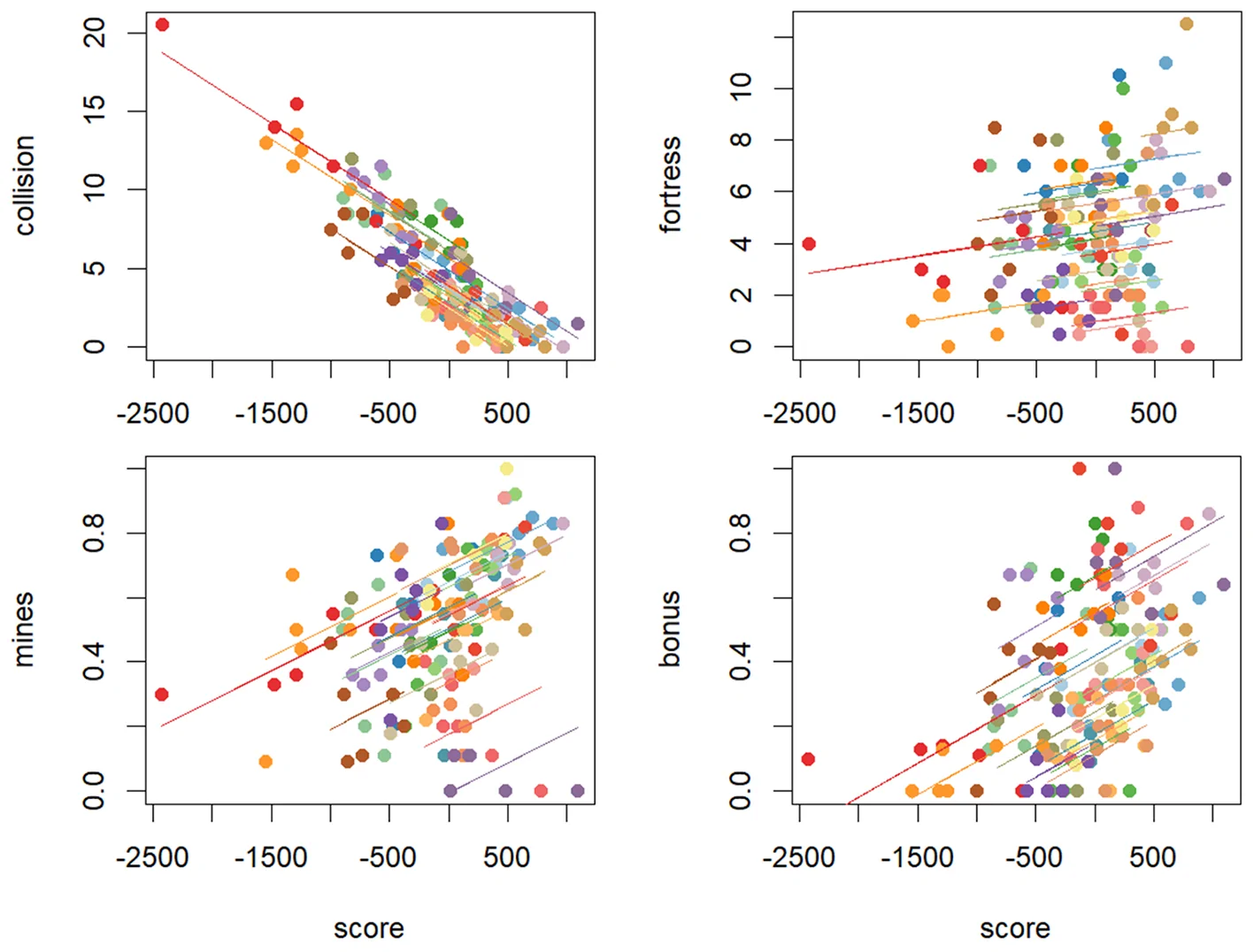
Repeated-measures correlations between overall SFG score and sub-scores. Top left: score ~ CPM; top right: score ~ FPM; bottom left: score ~ MD; bottom right: score ~ BC.

### 3.3 Prediction of SFG performance via EEG parameters

Based on the results of the earlier analyses, we decided to compute regression models only for the first and last SFG game (games one and six) and to only include EEG data 1- and 3-back data. Table 5 depicts the final predictors per model after stepwise elimination as well as the model fit. Figure 3 and Figure 4 depict the predictors of each model with their estimates and 95% confidence intervals. Statistical values for each predictor can be found in Table 6.

**Table 5:** Regression models with model estimates after stepwise elimination.

Prediction	Regression model	Predictors	*F*	*R^2^*	*R^2^ (adj.)*	*p*
Training—different task	SFG 1 score ~ EEG 1-back	Theta F3, theta F4, alpha P4, beta Pz	4.21	0.445	0.340	0.012
	SFG 1 score ~ EEG 3-back	Theta F3, theta F4, alpha P3, beta Pz	3.24	0.381	0.263	0.032
	SFG 6 score ~ EEG 1-back	Alpha P4, beta Pz, beta P3, beta P4, EI	4.45	0.527	0.409	0.007
	SFG 6 score ~ EEG 3-back	Theta Fz, alpha P4, beta Pz, beta P3, beta P4, TLI, EI	3.07	0.544	0.367	0.026
Training—same task	SFG 6 score ~ EEG SFG 1	Theta F3, theta F4, alpha Pz, alpha P3, beta Pz, beta P4, TLI, EI	3.31	0.609	0.425	0.018
Operator state—concurrent	SFG 1 score ~ EEG SFG 1	Theta Fz, theta F3, alpha P3, beta Pz	6.60	0.557	0.473	0.001
	SFG 6 score ~ EEG SFG 6	Theta F3, alpha Pz, alpha P3, beta Pz, beta P3, beta P4, TLI	4.73	0.648	0.511	0.004

**Figure 3:**
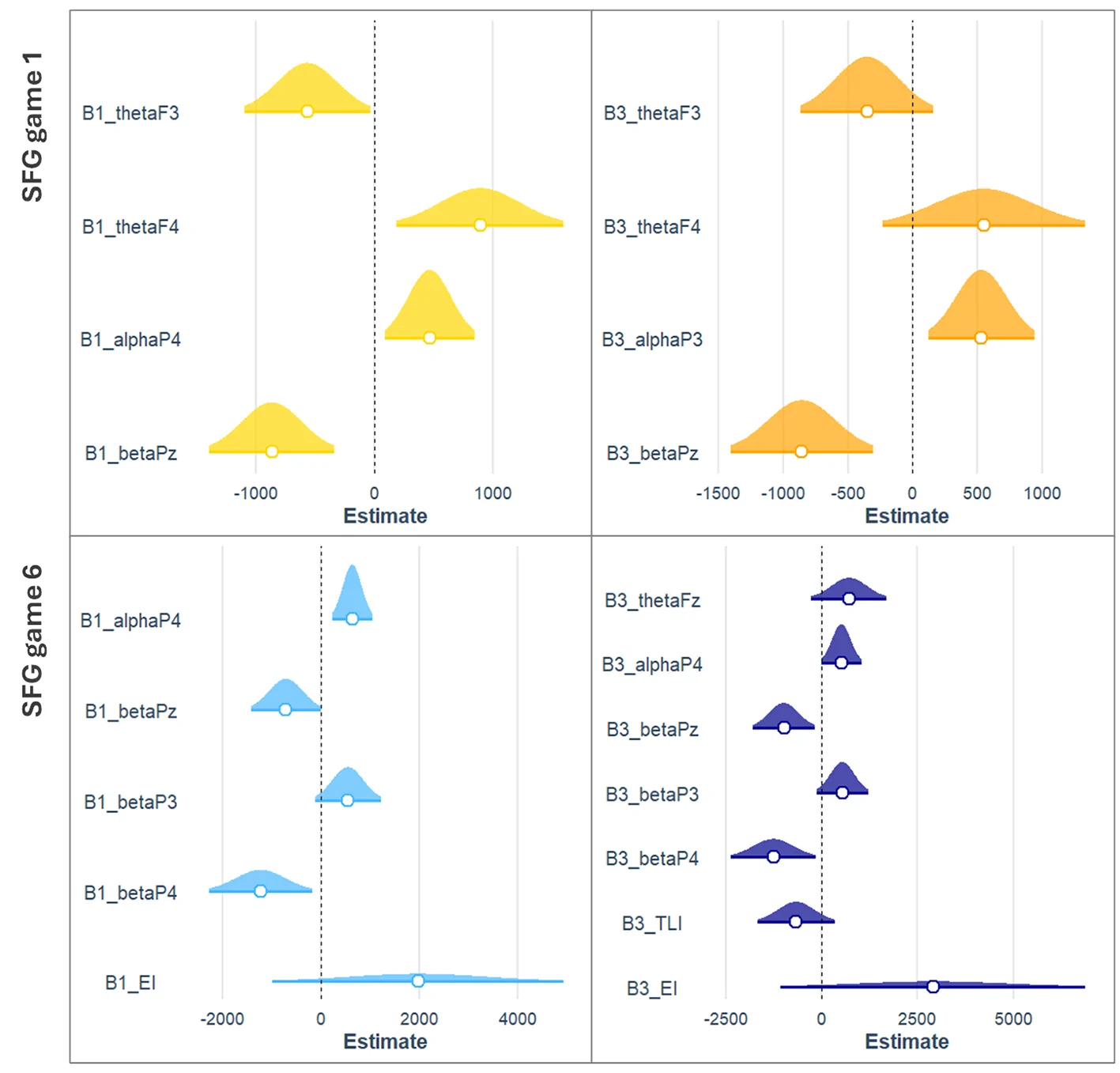
Predictors of the models for estimating the SFG score from the EEG data of the n-back task. From top left to bottom right: game 1 via 1-back, 3-back; game 6 via 1-back, 3-back. Circles indicate the estimate of the predictor, lines with bell curves indicate the 95% confidence interval.

**Figure 4:**
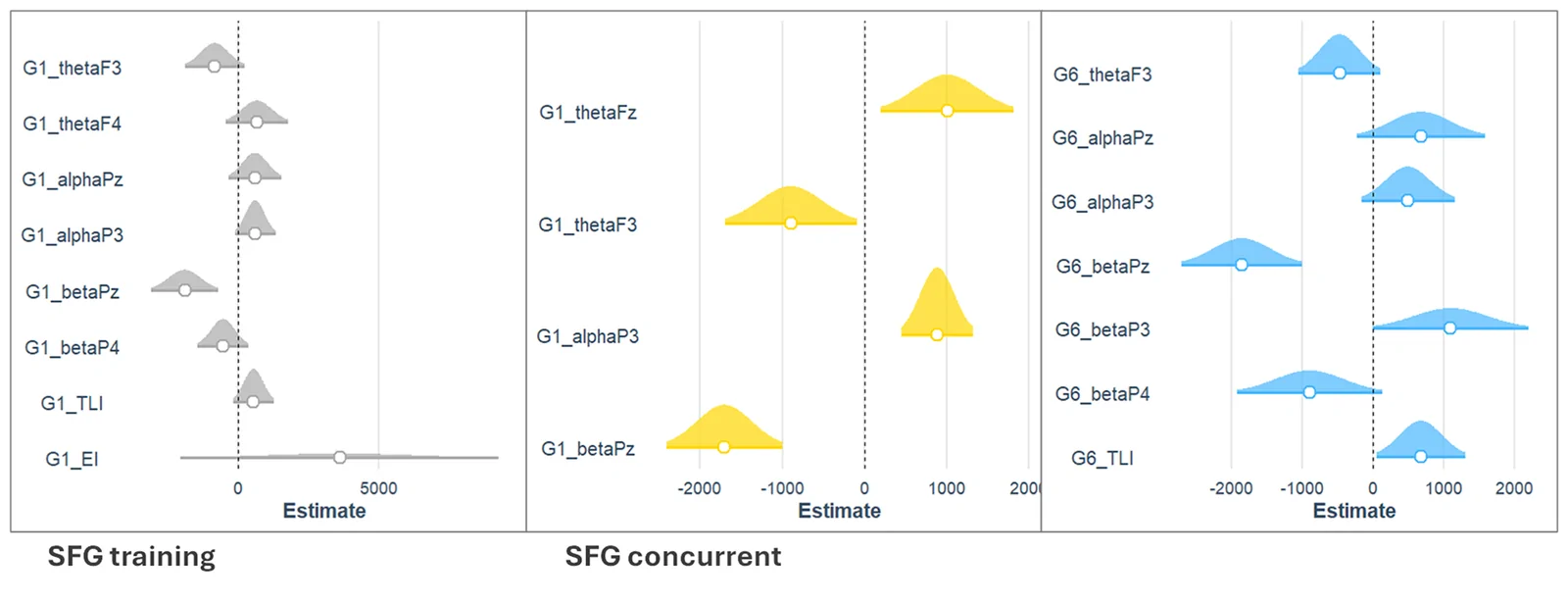
Predictors of the models for estimating the SFG score from the EEG data of previous and concurrent SFG games. From left to right: score game 6 via EEG game 1; score game 1 via EEG game 1; score game 6 via EEG game 6. Circles indicate the estimate of the predictor, horizontal lines with bell curves indicate the 95% confidence interval.

**Table 6:** Coefficients of the seven regression models.

Model	Coefficient	Estimate	*SE*	*t*	*p*
SFG 1 score ~ EEG 1-back	Intercept	−130.5	456.0	−0.29	0.777
	Theta F3	−564.7	255.4	−2.21	0.038^*^
	Theta F4	890.4	338.2	2.63	0.016^*^
	Alpha P4	465.6	182.5	2.55	0.019^*^
	Beta Pz	−863.7	254.5	−3.39	0.003^*^
SFG 1 score ~ EEG 3-back	Intercept	−15.9	480.9	−0.03	0.974
	Theta F3	−351.5	246.0	−1.43	0.168
	Theta F4	552.4	375.5	1.47	0.156
	Alpha P3	531.5	198.3	2.68	0.014^*^
	Beta Pz	−855.6	264.9 8	−3.23	0.004^*^
SFG 6 score ~ EEG 1-back	Intercept	681.9	469.8	1.45	0.162
	Alpha P4	640.6	195.1	3.28	0.004^*^
	Beta Pz	−710.1	342.6	−2.07	0.051
	Beta P3	551.8	321.9	1.72	0.102
	Beta P4	−1,225.9	504.6	−2.43	0.025^*^
	EI	1,978.7	1,420.8	1.39	0.179
SFG 6 score ~ EEG 3-back	Intercept	−212.8	943.3	−0.23	0.824
	Theta Fz	719.9	467.5	1.54	0.141
	Alpha P4	517.3	247.0	2.09	0.051
	Beta Pz	−980.5	388.1	−2.53	0.021^*^
	Beta P3	543.1	315.6	1.72	0.102
	Beta P4	−1,253.9	530.2	−2.37	0.030^*^
	TLI	−660.5	479.5	−1.38	0.185
	EI	2,903.8	1,886.3	1.54	0.141
SFG 6 score ~ SFG 1 score	Intercept	573.0	806.6	0.71	0.487
	Theta F3	−806.7	494.1	−1.63	0.121
	Theta F4	686.6	529.5	1.30	0.212
	Alpha Pz	616.9	451.2	1.37	0.189
	Alpha P3	621.7	340.2	1.83	0.085
	Beta Pz	−1,872.2	567.9	−3.30	0.004^*^
	Beta P4	−513.4	424.9	−1.21	0.243
	TLI	567.0	345.8	1.64	0.119
	EI	3,616.5	2,671.2	1.35	0.193
SFG 1 score ~ SFG 1 score	Intercept	633.4	440.5	1.44	0.165
	Theta Fz	1,003.5	389.2	2.58	0.018^*^
	Theta F3	−896.4	385.9	−2.32	0.030^*^
	Alpha P3	884.6	210.4	4.21	<0.001^*^
	Beta Pz	−1,704.5	338.7	−5.03	<0.001^*^
SFG 6 score ~ SFG 6 score	Intercept	1,305.2	449.1	2.91	0.009^*^
	Theta F3	−469.3	277.2	−1.69	0.108
	Alpha Pz	682.7	429.6	1.59	0.129
	Alpha P3	497.5	314.3	1.58	0.131
	Beta Pz	−1,851.5	407.9	−4.54	<0.001^*^
	Beta P3	1,101.2	525.5	2.10	0.051
	Beta P4	−893.6	487.8	−1.83	0.084
	TLI	681.5	299.2	2.28	0.035^*^

Significance threshold α = 0.05. Significant values marked ^*^.

Performance predictions based on the n-back task were better for the last compared to the first game (53 and 54 % vs. 38 and 45 % explained variance). The score of game one could be better predicted by EEG data from 1-back, whereas the predictions for game six did not differ much between 1- and 3-back. The model fit further increased for performance predictions from game one to game six (61 % explained variance). Concurrent performance predictions from EEG data of the same game were better for game six than game one (65 vs. 56 % explained variance).

Overall, the model fit estimates were better for predictions of game six than game one. The models for game one tended to be more parsimonious than those for game six, with fewer predictors remaining after elimination. Scores of the game one could be predicted mainly by theta, but also alpha and beta band activity. Scores of game six could be predicted mainly by beta band activity. The indices TLI and EI remained as predictors only in the models for game six.

## 4 Discussion

In the present study, we wanted to investigate whether performance in a complex cognitive task could be predicted (albeit in offline analyses, not in real-time) by brain activity from previous runs of the same task and from less complex tasks (predictive value of training) as well as from concurrent brain activity during task completion (concurrent operator state monitoring). For this purpose, we collected and analyzed EEG data from a dual n-back task that served as a low-complexity cognitive task and the SFG task, a well-known paradigm for training and skill acquisition research.

The EEG parameters behaved mostly as expected. In the n-back task, we observed the typical mental-workload associated pattern of increasing frontal theta and decreasing parietal alpha and beta activity with increasing task difficulty (Borghini et al., 2014), as well as a slight effect of training between runs one and two, i.e., increasing parietal alpha and beta activity and increasing task performance between the two runs. The only significant interaction between task difficulty and run manifested in the NASA-TLX ratings: subjective mental workload increased with task difficulty, and only decreased with run in the 2-back condition. Descriptively, the same pattern could be found for performance, i.e., a slight increase in performance in the 2-back condition between the runs. It is possible that this was due to training or a shift in strategy, and similar effects may have been masked by floor and ceiling effects in the other two n-back conditions, but since strategy use was not assessed, a deeper investigation was not possible.

In order to reduce complexity of the results presented in this paper, we thus focused on the first runs of the 1- and 3-back tasks for further analyses. In the SFG task, we observed lower variation in the EEG parameters, as expected with constant task difficulty. The slight increase in parietal alpha activity over time and the decrease in the TLI could serve as indicators of lower demand and thus successful training over time. Indeed, previous research shows that alpha activity plays an important role in SFG performance (Maclin et al., 2011; Mathewson et al., 2012).

The overall SFG score was highly associated with the participants' ability to control their ship, and moderately with two of the sub-tasks. This indicates that psychomotor skills influence the overall score more than the cognitive aspects of the game. Alternatively, our participants may have had less opportunity to engage with the cognitive aspects of the game because the duration of one game was rather short, or because the cognitive aspects of the game only gain relevance to the players once their training is more advanced and they have free cognitive resources to spend elsewhere than the ship steering (Logie et al., 1989). In addition, the game duration and scoring of the SFG sub-tasks is variable and highly influences the overall score. This can be seen from the non-significant association between the overall score and hits on the fortress, theoretically the players' most important goal in the game. In our experiment, each game lasted only two min, which made it rather difficult to destroy the fortress and rarely resulted in points awarded for this task. Thus, destroying the fortress did not influence the overall score as much as the other sub-tasks. The players may have adapted their strategies accordingly and prioritized ship control, destroying mines and capturing bonuses over destroying the fortress in order to gain as many points as possible. Because of that, in our configuration of the game, the overall score can be seen as the most representative indicator of overall game performance. This aspect is relevant for all studies using the SFG. The paradigm is intended for flexible use as all parameters can be configured freely and tasks can be removed completely if this serves the purpose of the study (Chenot et al., 2021). Nevertheless, the overall score is the most salient performance measure for the participants as it is by default displayed continuously on the top of the screen as well as shown prominently after each game. Thus, the association between sub-tasks, scoring, instruction and participants' strategy use should be taken into account when interpreting data.

The regression analysis showed that it is possible to predict complex task performance in the SFG task from EEG data. All seven computed models explained the variance in the data better than the null model. While the model fit in all models was moderate, with a maximum of 65 % explained variance, the results are comparable to those of previous fMRI-based (55 % pre- vs. post-training; Vo et al., 2011) and EEG-based (58 % early vs. late training; Mathewson et al., 2012) predictions of SFG scores and surpass those of fNIRS-based predictions (32 % on-task brain activity, 26 % resting state brain activity; Chenot et al., 2021). As an important side note, most models contained one or multiple predictors that did not meet the conventional significance threshold of *p* = 0.05. This is an artifact of using the AIC as a criterion for model evaluation instead of removing parameters based on their *p*-value. (for a thorough explanation see the discussion of “confusing” parameters in Sutherland et al., 2023). Thus, in the following, we discuss overall model prediction accuracy, but interpret single predictors only with caution.

The prediction accuracy increased from the dissimilar cognitive task to the same cognitive task and was highest for concurrent EEG data during task completion. This pattern is not surprising because similar tasks should cause similar brain activity, thus increasing the predictive value of the data. Prediction accuracy seems to increase with increasing on-task training. The models for game one tended to be more parsimonious than those for game six, with fewer predictors remaining after elimination. Yet, the model estimates were better for SFG game six than game one, especially for the predictions based on the n-back tasks. Due to the sample size of *N* = 26, we cannot rule out over-fitting the models, and our results should thus be interpreted carefully. Future research should aim at replicating our findings with a larger sample and ideally attempt cross-validation with a second independent sample to increase confidence in the robustness and generalizability of the models across samples.

A second explanation for better model fit in the later game is training and strategy stability. It is possible that task performance in game one was still too variable to enable good predictions. Participants were still focusing on steering the ship and the cognitive and strategic components of the game like memorizing mine designators or bonus indicators likely factored more into the later games (Towne et al., 2016). This aligns with the finding that the clearly working-memory related dual n-back task explained more variance in the last compared to the first SFG game. Working memory and multitasking seem quite important aspects of the SFG (Logie et al., 1989). This indicates that complex task performance could be inferred from less complex training tasks as long as they contain the most important aspects of the complex task. This could help improve training efficiency by incorporating part-task training or simplified tasks into the schedule as intermediate steps or checks if the realistic task is difficult to train or particularly time-consuming and costly. Future research in this regard is certainly needed, but seems promising based on our current findings.

The best model fit was achieved for concurrent EEG data predicting task performance. This supports the feasibility of operator state monitoring, i.e., utilizing real-time physiological data to monitor operators' cognitive states and performance. Our findings also indicate that performance in a complex task cannot be explained by single predictor, but that multiple frequency bands and measurement locations are necessary to achieve a good model fit. This may also explain why the explained variance in our models was greater than that in previous research using fNIRS (Chenot et al., 2021). The authors used only one predictor, an average Pearson's correlation coefficient for all measured channels and only for oxygenated blood. Our present results indicate that it might be useful to include multiple predictors instead and eliminate those that do not add to the model. Yet, further research is necessary in order to investigate whether this pattern can be replicated and which predictors consistently improve regression models across studies.

In line with the prediction accuracy, the EEG parameters that remained in the models after stepwise elimination varied between SFG games one and six. Contrary to previous findings (Mathewson et al., 2012), alpha activity could only be considered a good predictor for early SFG performance (game one), but seldom remained in the models in game six. The same pattern could be observed for frontal theta power that seemed rather important in all three models for game one, but less so for game six. This pattern is in line with the literature on mental workload: frontal theta and parietal alpha activity are prominent features in explaining mental workload from EEG data (Holm et al., 2009; Borghini et al., 2014; Hamann and Carstengerdes, 2022). Even after the practice, participants likely still learned and adapted their strategies on-task and thus experienced higher cognitive demand in the first compared to the last game.

Parietal beta activity seemed to gain importance from game one to game six. Interestingly, beta activity at Pz was the only EEG parameter that remained in all regression models. Recent research suggests that parietal and posterior beta power suppression plays an important part in task switching during working memory tasks (Myers et al., 2026) and that reduced beta power seems to be associated with clearing no longer needed information from working memory tasks (Lundqvist et al., 2018, 2024; Myers et al., 2026). It is possible that the effects we observed rely on the same mechanisms since our experimental tasks required frequent updates of working memory items and task switching as well. This may suggest that parietal beta activity, especially at Pz, may be a good candidate for performance prediction in working memory tasks. However, given the small sample size of this study, this result needs further investigation.

Similar to beta activity, the indices TLI and EI became more prominent in explaining variance in game six. Since the EI contains substantial parietal beta activity, this similar trend could be expected. However, importantly, if the EI remained as a predictor in a model, it seemed to pool residual variance, as can be seen from the broad confidence intervals. The TLI, on the other hand, contains only alpha and theta activity. Its prominence in the later game is somewhat surprising. Indeed, previous research has cast doubt on the utility of the two indices for assessing mental workload because of their *post-hoc* nature and the bundling of multiple electrodes and bands that complicates the interpretation of the changes and their origins (Hamann and Carstengerdes, 2023). This is the main reason we wanted to further test their utility in the present study. Here, the indices added to the regression models on top of the single electrodes and bands. However, the indices contain different bandwidths for the alpha, beta and theta bands than the single electrodes (Pope et al., 1995; Smith et al., 2001) as well as (for the EI) more electrodes (Pope et al., 1995). It is possible that the addition of single electrodes with varying bandwidths to the models would result in elimination of the indices, but we did not test this in this study. This discussion underlines that the choice of EEG parameters for analysis is not an easy one. Electrodes, their combination into regions of interest or indices and variations in the specific frequency bands and extracted metrics can influence the results. Thus, researchers should pay attention to specify the metrics they employed as detailed as possible in order to foster transparency and interpretability of the results.

In conclusion, we could show that it is possible to predict complex task performance from brain activity during training, both from same-task training and, interestingly, from less complex tasks. Especially the latter results are of interest for designing and tailoring simulator trainings for operators with highly demanding, complex tasks such as pilots and UAV operators. Including less complex training tasks or part-task training could make training schedules more flexible, cost-efficient and tailored to the operators' needs, thus adding to current approaches of competency-based training schedules (Kearns et al., 2016). Moreover, we could predict performance from concurrent brain activity, an important step toward operator state monitoring. Our results also show the importance of multiple linear regression models to explain complex task performance. Since we worked with offline-analyses only, future research should expand this approach to real-time predictions during task performance. Nevertheless, our findings thus add to the body of research on the SFG as a training tool and the state of the art in operator state monitoring.

## Data Availability

The raw data supporting the conclusions of this article will be made available by the authors, without undue reservation.
