# Supplementary material for: I think, therefore I hit: EEG parameters predict performance in complex cognitive tasks
Source: Front Neuroergon. 2026 Sep 23;7:1894934. doi: 10.3389/fnrgo.2026.1894934 (PMC13642727; doi:10.3389/fnrgo.2026.1894934)
Supplement: Supplementary file 1 [file Data_Sheet_1.pdf]

Supplementary Material for the Article "I Think, Therefore I Hit: EEG Parameters Predict Performance in Complex Cognitive Tasks"

by Anneke Hamann, Julia Schön & Nils Carstengerdes

Table S1. Results of the post-hoc pairwise comparisons for the n-back task.

Parameter	N-back level (t, p-value)			Run (t, p-value)
	1 vs. 2	1 vs. 3	2 vs. 3	1 vs. 2
Theta Fz	-3.47 (.002*)	-1.64 (.233)	1.83 (.165)	-1.48 (.142)
Theta F3	-0.89 (.648)	0.63 (.803)	1.52 (.284)	-1.22 (.223)
Theta F4	-0.77 (.725)	1.91 (.142)	2.67 (.023*)	1.01 (.314)
Alpha Pz	2.23 (.071)	2.80 (.016*)	0.57 (.835)	-3.81 (< .001*)
Alpha P3	3.00 (.009*)	2.58 (.030*)	-0.43 (.905)	-3.21 (.002*)
Alpha P4	2.76 (.018*)	3.16 (.006*)	0.41 (.912)	-2.97 (.004*)
Beta Pz	5.42 (< .001*)	5.74 (< .001*)	0.32 (.946)	-3.36 (.001*)
Beta P3	2.68 (.022*)	2.44 (.043*)	-0.25 (.967)	-1.12 (.265)
Beta P4	1.45 (.319)	1.74 (.196)	0.29 (.956)	-2.08 (.040*)
TLI	-2.63 (.026*)	-1.69 (.213)	0.94 (.615)	0.15 (.879)
EI	3.60 (.001*)	1.98 (.121)	-1.62 (.241)	-0.62 (.535)
NASA-TLX	-8.66 (< .001*)	-12.79 (< .001*)	-4.18 (.002*)	1.04 (.300)
% correct	9.39 (< .001*)	20.22 (< .001*)	10.94 (< .001*)	-2.27 (.025*)

Note: Significance threshold $\alpha = .05$, Tukey-corrected. Significant comparisons marked *.

Table S2. Results of the post-hoc pairwise comparisons for the SFG task.

Parameter	Game (*t*, *p*-value)														
	1 vs. 2	1 vs. 3	1 vs. 4	1 vs. 5	1 vs. 6	2 vs. 3	2 vs. 4	2 vs. 5	2 vs. 6	3 vs. 4	3 vs. 5	3 vs. 6	4 vs. 5	4 vs. 6	5 vs. 6
Alpha Pz	-2.66 (.092)	-2.64 (.095)	-3.49 (.009*)	-2.48 (.139)	-3.37 (.013*)	0.01 (1.000)	-0.83 (.961)	0.18 (1.000)	-0.71 (.980)	-0.85 (.958)	0.17 (> .999)	-0.73 (.978)	1.01 (.913)	0.12 (> .999)	-0.90 (.947)
Alpha P3	-1.86 (.433)	-3.10 (.028*)	-3.16 (.024*)	-2.55 (.118)	-1.98 (.363)	-1.24 (.815)	-1.30 (.786)	-0.69 (.982)	-0.12 (> .999)	-0.06 (> .999)	0.55 (.994)	1.13 (.870)	0.60 (.991)	1.18 (.846)	0.58 (.992)
TLI	-1.73 (.518)	1.40 (.727)	-0.77 (.972)	0.46 (.997)	-0.63 (.989)	3.13 (.026*)	0.96 (.931)	2.19 (.250)	1.10 (.882)	-2.17 (.259)	-0.94 (.936)	-2.03 (.332)	1.23 (.820)	0.14 (> .999)	-1.09 (.883)
Score	0.67 (-.985)	-0.54 (.994)	-1.46 (.691)	-2.33 (.191)	-2.77 (.070)	-1.21 (.830)	-2.13 (.278)	-3.00 (.038*)	-3.44 (.010*)	-0.92 (.941)	-1.79 (.478)	-2.23 (.234)	-0.87 (.953)	-1.31 (.782)	-0.44 (.998)
CPM	-0.81 (.965)	-0.32 (>.999)	0.99 (.920)	1.27 (.799)	1.45 (.696)	0.50 (.996)	1.80 (.467)	2.09 (.301)	2.26 (.217)	1.31 (.780)	1.59 (.606)	1.77 (.490)	0.28 (> .999)	0.46 (.997)	0.18 (> .999)
FPM	-1.21 (.832)	-1.30 (.787)	-2.55 (.118)	-3.80 (.003*)	-3.24 (.019*)	-0.09 (> .999)	-1.34 (.763)	-2.59 (.107)	-2.03 (.332)	-1.25 (.810)	-2.51 (.131)	-1.94 (.381)	-1.25 (.810)	-0.69 (.983)	0.56 (.993)
MD	0.63 (.988)	-0.25 (> .999)	-1.08 (.890)	-2.23 (.230)	-1.91 (.399)	-0.88 (.950)	-1.71 (.529)	-2.87 (.054)	-2.55 (.119)	-0.83 (.962)	-1.99 (.357)	-1.67 (.558)	-1.16 (.856)	-0.84 (.960)	0.32 (> .999)
BC	-1.13 (.868)	-0.95 (.932)	-2.02 (.336)	-2.76 (.071)	-3.55 (.007*)	0.18 (> .999)	-0.89 (.948)	-1.63 (.580)	-2.42 (.156)	-1.07 (.893)	-1.81 (.464)	-2.60 (.105)	-0.74 (.977)	-1.53 (.644)	-0.79 (.968)

Note: Significance threshold α = .05, Tukey-corrected. Significant comparisons marked *.
